# The role of NAD-dependent deacetylase sirtuin-2 in liver metabolic stress through regulating pyruvate kinase M2 ubiquitination

**DOI:** 10.1186/s12967-024-05435-w

**Published:** 2024-07-14

**Authors:** Jingru Guo, Junshu Nie, Dongni Li, Huaixiu Zhang, Tianrui Zhao, Shoufeng Zhang, Li Ma, Jingjing Lu, Hong Ji, Shize Li, Sha Tao, Bin Xu

**Affiliations:** 1 National Experimental Teaching Demonstration Center of Animal Medicine Foundation, College of Animal Science and Veterinary Medicine, Heilongjiang Bayi Agricultural University, Daqing, China; 2 The University of Georgia, Athens, GA USA

**Keywords:** Sirt2, Acetylation, PKM2, Glycolysis, Metabolism

## Abstract

NAD-dependent deacetylase Sirt2 is involved in mammalian metabolic activities, matching energy demand with energy production and expenditure, and is relevant to a variety of metabolic diseases. Here, we constructed *Sirt2* knockout and adeno-associated virus overexpression mice and found that deletion of hepatic Sirt2 accelerated primary obesity and insulin resistance in mice with concomitant hepatic metabolic dysfunction. However, the key targets of Sirt2 are unknown. We identified the M2 isoform of pyruvate kinase (PKM2) as a key Sirt2 target involved in glycolysis in metabolic stress. Through yeast two-hybrid and mass spectrometry combined with multi-omics analysis, we identified candidate acetylation modification targets of Sirt2 on PKM2 lysine 135 (K135). The Sirt2-mediated deacetylation-ubiquitination switch of PKM2 regulated the development of glycolysis. Here, we found that Sirt2 deficiency led to impaired glucose tolerance and insulin resistance and induced primary obesity. Sirt2 severely disrupted liver function in mice under metabolic stress, exacerbated the metabolic burden on the liver, and affected glucose metabolism. Sirt2 underwent acetylation modification of lysine 135 of PKM2 through a histidine 187 enzyme active site-dependent effect and reduced ubiquitination of the K48 ubiquitin chain of PKM2. Our findings reveal that the hepatic glucose metabolism links nutrient state to whole-body energetics through the rhythmic regulation of Sirt2.

**Supplementary Information:**

The online version contains supplementary material available at 10.1186/s12967-024-05435-w.

## Introduction

Sirtuin, the human and mouse homolog of the yeast silent information regulatory gene, is an NAD^+^-dependent deacetylase. Sirt2 is metabolically active and highly enriched in tissues, including liver, heart, brain, and adipose tissue, and plays a regulatory role in maintaining metabolic homeostasis and aging [1]. Studies have shown that Sirt2 deacetylates glucokinase regulatory proteins and promotes impaired levels of hepatic glucose [2]. Epidemiological studies have shown that the prevalence of metabolic diseases, including diabetes mellitus type 2 (T2DM), has increased significantly worldwide and that dysglycemia and insulin resistance are the main responses to the development of T2DM [3]. In T2DM, Sirt2 overexpression significantly enhances the activation of AKT and its downstream targets involved in insulin resistance [4]. As a deacetylase, Sirt2 maintains energy homeostasis by deacetylating Foxo1 and inhibiting PPARγ to promote lipolysis [5]. Both the Sirtuin family and Sirt2 genes can sense the nutritional requirements and availability of an organism and optimize cellular pathways to match energy production with consumption [6–10], with feedback effects on the glycolytic pathway [11–13]. Mice deficient in SIRT2 develop liver tumors [14, 15], and exhibit loss of metabolism and increased glucose metabolism.

It is known that ambient temperature also affects mammalian energy metabolism, and when the ambient temperature is low, metabolic reactions increase energy expenditure by using glucose and lipids from adipose tissue and the liver through shivering and non-shivering thermogenesis [16]. This temperature change-induced stress leads to metabolic stress, a state of metabolic disturbances such as elevated blood glucose and elevated levels of hormones. Usually, low temperature induces a state of cold acclimation and a new equilibrium is reached after altering stress hormone levels and other physiological responses [17]. However, current conclusions related to the effects of hypothermia on the body’s glucose metabolism are also very inconsistent. Transient hypothermia can significantly increase energy expenditure, increase plasma glucose oxidation and insulin sensitivity, and accelerate systemic glucose metabolism, which can affect the body’s energy metabolism leading to lower blood glucose levels [18–20]. However, one study observed that acute exposure of rats to hypothermia within 1 week resulted in decreased insulin levels, increased insulin sensitivity in peripheral tissues, and sustained high levels of glucagon with blood glucose remaining unchanged [21, 22].

Pyruvate kinase, one of the major rate-limiting enzymes in glycolysis, catalyzes the production of pyruvate and ATP from phosphoenolpyruvate and determines ATP production through oxidative phosphorylation or glycolytic intermediates. PKM2 is an isoform of PK, and the function of PKM2 is dependent on post-translational modifications such as acetylation, phosphorylation, and sumo-ization [23, 24]. Unlike other isoforms, PKM2 possesses a large number of conserved post-translational modification sites. Acetylation of Lys305 reduces the enzymatic activity of PKM2 and degrades PKM2 in an autophagy-lysosome-dependent manner, ultimately stimulating the Warburg effect and tumor growth. High expression levels of PKM2 lead to increased glucose uptake, accumulation of glycometabolites and metabolic reprogramming (from oxidative phosphorylation-based to glycolysis-based). And PKM2 plays an important role in tumor metabolic reprogramming, acetylation modification can regulate PKM2 function and affect energy metabolism. However, the mechanism underlying this lysine acetylation remains unclear. As a protein kinase or transcriptional co-activator, PKM2 is used for ATP production via oxidative phosphorylation when it is in the tetrameric state. Conversely, when PKM2 is dissociated, a decrease in enzymatic activity is observed that prevents pyruvate production. This leads to increased synthesis of macromolecules, including lipids, nucleic acids, and amino acids [25]. It has been found that SIRT2 can interact with PKM2 and affect the metabolic reprogramming and proliferation of tumor cells by regulating the interaction between PKM2 and SIRT2, providing new targets and strategies for tumor therapy. SIRT2 modifies PKM2 through deacetylation, affecting the activity and stability of PKM2, which in turn affects intracellular glycolytic processes [26]. In addition to being a key enzyme in glycolysis, PKM2 is involved in multiple signaling pathways. Activation or inhibition of some signaling pathways can affect glycolysis by regulating the activity or conformational state of PKM2, thereby regulating cellular energy metabolism and growth. Overall, there are complex interactions and regulatory relationships between PKM2 and SIRT2. By studying the function and regulatory mechanism of PKM2 in glycolysis, as well as the interaction between SIRT2 and PKM2, we can help to reveal the fundamentals of cellular energy metabolism regulation and provide new ideas and strategies for the treatment of related diseases.

Therefore, this experiment will construct a model of metabolic stress in the organism by intermittent hypothermia to help elucidate the glucose metabolic processes occurring in the organism. In this study, we constructed *Sirt2* knockout mice and adeno-associated virus overexpression mice, and revealed PKM2 interacted with Sirt2 using a yeast two-hybrid system and proteomics. By combined multi-omics analysis, we identified previously unknown acetylation modification sites of PKM2. Furthermore, we found that deletion of Sirt2 led to impaired glucose tolerance and insulin resistance, and induced primary obesity; Sirt2 severely disrupted liver function in mice under metabolic stress, exacerbating the metabolic burden on the liver and affecting glucose metabolism. Sirt2 deacetylated PKM2-Lys135 (K135) in a histidine 187 (H187) site-dependent manner and reduced ubiquitination of the Lys48 (K48) site ubiquitin chain of PKM2 thereby altering the adaptation to metabolic stress responses and ultimately regulating glycolysis.

## Materials and methods

### Animals

Mice were placed in a temperature/humidity-controlled environment (24 ± 2 °C/40%) and were maintained on a 12 h light/12 h dark cycle (8 a.m. and 8 p.m.) with free access to food and water. C57BL/6 mice were purchased from the Changsheng Biological Corporation (Changchun, China) and randomly divided into two groups: a cold exposure group and a room temperature control group. The cold exposure group was exposed to a stimulation temperature of 4℃ and humidity of 40% for 3 h per day for 3 weeks. All groups in one experiment contained individuals of the same strain and sex. C57BL/6 mice were used for long-term experiments. *Sirt2* knockout mice were generated on a C57BL/6 background. The *Sirt2* gene (NCBI Reference Sequence: NM_022432; Ensembl: ENSMUSG00000015149) is located on mouse chromosome 7. Sixteen exons are identified, with the ATG start codon in exon 1 and the TAA stop codon in exon 16 (Transcript Sirt2-201: ENSMUST00000072965). Exon 5 ~ 7 will be selected as target site. The region contains 206 bp coding sequence. Ribonucleoprotein (RNP) will be co-injected into fertilized eggs for KO mouse production. The pups will be genotyped by PCR followed by sequencing analysis. All experimental procedures were approved by the Management Committee of Laboratory Animal Center of Heilongjiang Bayi Agricultural University.

### Adeno-associated virus 9 (AAV9)-mediated gene expression

First, the target gene was cloned into the AAV plasmid with appropriate identification and validation. Then, mass extraction of the plasmid was carried out using a de-endotoxin kit AAV plasmid system co-transfected AAV-293 cells, and the AAV plasmid was replicated and assembled into complete AAV virus particles in the cells by the Rep and Cap proteins provided by the helper viruses, and all the cells were collected at 72 h post-transfection. Finally, the cell culture supernatant was purified by centrifugation and column chromatography to obtain a high-purity AAV virus preparation. The AAV9 delivery system was used for liver-specific overexpression of *Sirt2* in mice. The open reading frame encoding these genes, without stop codons, was cloned into the AAV9 vector AAV-pTBG-Luciferase. Each mouse was injected with 100 µl of AAV9 virus solution containing the target gene via tail vein at a titer of 1.4 × 10^12^ vg/mL. Target gene expression was monitored by bioluminescence imaging (BLI) after 3 weeks.

### Cell culture and treatment

Mouse AML-12 hepatocytes were cultured in DMEM/F12 medium supplemented with 10% fetal bovine serum (FBS), 40 ng/mL Dexamethasone, and ITS liquid media supplement. HEK293T cells were cultured in DMEM supplemented with 10% FBS and antibiotics at 37 °C and 5% CO_2_ in a humidified incubator. AML12 cells were treated for 12 h with 300 µM CoCl2 (Sigma-Aldrich, St. Louis, MO, USA; C8661).

### Histopathological analysis

Oil Red O, hematoxylin and eosin (H&E), and Masson staining were performed on serial Sect. (5 μm thick) of liver tissue specimens. The sections were observed under an optical microscope.

### Immunofluorescence (IF) staining

For immunofluorescence staining, cells were grown on microscope cover glasses and treated as indicated. After treatment, the cells were fixed with 4% paraformaldehyde, blocked with 0.2% Triton X-100 for 15 min and incubated with antibodies against Sirt2 (Proteintech, Rosemont, IL, USA; 19655-1-AP) and PKM2 (Cell Signaling Technology, Danvers, MA, USA; 4053) overnight for approximately 12 h at 4 °C. The cells were then washed with phosphate-buffered saline (PBS) (Beyotime, Beijing, China; C0221A) and incubated with fluorescently labeled secondary antibodies for 1 h at room temperature; 4ʹ,6-diamidino-2-phenylindole (DAPI) (Beyotime; C1005) was used to stain nuclei. Sections were observed under a confocal microscope (Nikon, Tokyo, Japan).

### Immunohistochemistry (IHC) staining

Sections were stained with PKM2 (1:200) primary antibody (Rabbit) followed by HRP antibody (Rabbit). A DAB Plus Substrate (Sigma-Aldrich) with added DAB Plus Chromogen was employed to visualize staining.

### Serology and glucose and insulin tolerance tests

Serum levels of ALT, AST, and TG were measured using a veterinary automatic biochemical analyzer (Seamaty, SMT-120VP). Mice were individually caged for 3 days before experiments. Mice were fasted for 16 h (from 5 p.m. to 9 a.m.) and blood glucose was measured through the tail vein using an automatic glucometer (Yicheng, city, country; JSP-6). Then, glucose was intraperitoneally injected at 1 g/kg body weight, and blood glucose levels were measured at various time points. For the ITT, mice were treated as in the GTT, except they were intraperitoneally injected with a single dose of insulin (1 U/kg). The mice used for the GTT and ITT assays were from different groups to prevent stress interference from earlier blood collection.

### Quantitative real-time PCR

Total RNA was isolated with TRIzol reagent (Invitrogen, Carlsbad, CA, USA). Purified RNA was retro-transcribed using the RT Kit (Takara, RR047A) and quantitative real-time PCR experiments were performed. All reactions were repeatedly processed. Primers used for quantitative real-time PCR were (5´-3´): mouse β-actin, GATGGCCACTGCCGCATCCTC and GGTCTTTACGGATGTCAACGTCAC; Sirt2, CTCATCAGCAAGGCACCACTAGC and CATCCGAGGAGGTCAGCGAGAG; and PKM2, AAGTTTACACGAAGGTCGACAT and TATCATTGCCGTGACTCGAAAT.

### Western blot analysis

Western blot analysis was performed as described previously[62]. Total protein (30 µg) was immunoblotted with rabbit anti-Sirt2 (Proteintech, 19655-1-AP, 1:1,000), rabbit anti-PKM2 (Proteintech, 19655-1-AP, 1:1,000; Cell Signaling Technology, 4053, 1:1,000), rabbit anti-ATP5a1, rabbit anti-UQCRC2, rabbit anti-SDHB, rabbit anti-NDUFB8, and mouse anti-actin (Proteintech, 66009-1-Ig, 1:10,000) followed by corresponding secondary antibodies conjugated to horseradish peroxidase (HRP) for 1 h [HRP-conjugated Affinipure goat anti-mouse IgG (H + L) (Proteintech, SA00001-1, 1:8,000) and HRP-conjugated Affinipure goat anti-mouse IgG(H + L) (Proteintech, SA00001-1, 1:8,000)].

### Seahorse bioenergetics

Primary mouse hepatocytes were isolated and cultured in Seahorse XF24 Extracellular Flux Analyser (Agilent Technologies, Santa Clara, CA, USA) culture plates with 2 × 104 cells per well. Plates were incubated for 1 h at 37 °C in an incubator without CO_2_ according to the instructions of the XF Cell Mito Stress Test Kit (103015-100; source and address) and a XF Glycolysis Stress Test Kit (103020-100; source and address). Oxygen consumption (OCR) and extracellular acidification rate (ECAR) were measured as instructed. Data were assessed with XF Wave Software. Liver blocks with a diameter of 3 mm were cut off and placed in tissue culture trays. OCR and ECAR were measured as instructed.

For liver tissue, inoculation was measured at the same weight on hippocampal tissue culture plates, and real-time mitochondrial respiration was measured on the hippocampal XFe24 analyzer using the Mitochondrial Stress Assay Kit.

### RNA sequencing

RNA sequencing was performed by Biomarker (Beijing, China). Briefly, RNA from liver tissue (20 mg) was extracted and quantified using a NanoDrop (ThermoFisher Scientific, Waltham, MA, USA) and used to construct RNA libraries that were sequenced using Illumina NovaSeqTM 6000 by Biomarker (Beijing, China).

### Targeted metabolomics

Liver tissue (100 mg) was weighed, 1 mL of pre-cooled methanol/acetonitrile/water was added, and the samples were left by ultrasound in an ice bath then centrifuged at 16,000 × g at 4℃ for 30 min. The supernatant was removed. The same amount of internal standard L-glutamate-D5 was added to each sample and vacuum dried. During mass spectrometry detection, 100 µL of acetonitrile-water solution was added for resolution, and the samples were centrifuged at 16,000 × g at 4℃ for 30 min and the supernatant removed for sample analysis. Separation was performed by Shimadzu Nexera X2 LC-30AD high performance liquid chromatography. Mass spectrometry was performed using a QTRAP5500 Mass Spectrometer (AB SCIEX, Redwood City, CA, USA) in positive/negative ion mode. The peak area and retention time were extracted by MultiQuant software. A metabolite standard was used to adjust retention time and identify metabolites. The peak area of metabolite extracted ions was normalized by the internal standard L-glutamate_D5. Encyclopedia of Genes and Genomes (KEGG) pathway enrichment analysis were applied for the differentially expressed related genes.

### Coimmunoprecipitation (Co-IP)

Co-IP were performed as described by Song. Briefly, liver tissues and cells were lysed in IP-lysis buffer [50 mM Tris (pH 7.4), 150 mM NaCl, 0.1 mM EDTA, 20% glycerol, 0.2% NP-40, 0.1% SDS, protease and phosphatase inhibitors]. After freezing and centrifugation at high speed, the lysate supernatants were incubated with the indicated antibodies at 4℃. The pre-cleared protein A/G beads (Thermo Fisher Scientific; 88,803) were then incubated with the supernatant at room temperature for 1.5 h. The collected proteins were analyzed by western blotting.

### Metabolic measurements

Mice were housed in metabolic cages individually with access to food and water in metabolic chambers of the Comprehensive Laboratory Animal Monitoring System to monitor global metabolism (CLAMS™, Columbus Instruments, Columbus, OH, USA). After acclimation for 24 h, energy expenditure was measured for 24 h. Metabolic parameters including carbon dioxide production (VCO_2_), oxygen consumption (VO^2^), respiratory exchange ratio (RER = VCO2/VO2), and heat production (heat) were recorded every 12 min over a period of 24 h and determined using an Oxymax system (Columbus Instruments).

### Oxidative stress detection in vivo

ROS generation was detected by dihydroethidium (DHE) staining. Briefly, frozen liver sections were incubated with DHE (5 µmol/L) at 37 °C for 30 min in a dark chamber. Fluorescent images were observed with a fluorescence microscope (Olympus IX53; Olympus, Tokyo, Japan).

### TdT-mediated dUTP nick end-labelling (TUNEL) staining

Liver Sect. (10 μm) were fixed with 4% paraformaldehyde for 45 min at room temperature and TUNEL staining was measured using a kit (Beyotime) according to the manufacturer’s instructions. Images were acquired using a confocal microscope (FV3000; Olympus).

### Cycloheximide and MG132 assays

CHX or MG132 was added to the culture medium at a final concentration of 10 µM or 20 mM, respectively. Cell lysates were collected at 0, 1, and 4 h after CHX or 4 h after MG132 treatment.

### Yeast two hybrid

Using the SIRT2 gene constructed on the pGBKT7 vector as bait, a yeast two hybrid mouse library was screened. After multiple reporter gene detection, DNA sequencing, and BLAST comparison analysis of positive clones, the proteins that interact with pGBKT7 SIRT2 were identified. Amplify positive clones from yeast cells for DNA sequencing and perform BLAST alignment analysis with sequences from the GenBank database. Identify positive cloned genes screened from SD-TLH + 5 mM 3AT plates. Dilute the positive clone transformants with sterile water and point them to SD-TL, SD-TLH + 5 mM 3AT, SD-TLHA + 5 mM 3AT, and SD-TLHA + 5 mM 3AT + X, respectively-α- Gal defect plate, incubated at 30 ℃ for 3–4 days.

### Detection of enzyme activity and content

Enzyme activity was detected using a Lacate Dehydrogenase Activity Assay Kit or a Pyruvate kinase Assay Kit (Solarbio Science and Technology, Beijing, China), following the manufacturer’s instruction. According to the ratio of the number of cells to the volume of the extracted liquid is 500 ~ 1000:1, ultrasonic crushing, standing for 30 min, 8000 g, centrifugation at room temperature for 10 min, and the supernatant is taken and tested according to the instructions; According to the ratio of liver tissue mass to the volume of extracted fluid is 1:5 ~ 10, the homogenize in an ice bath, stand for 30 min, 8000 g, centrifuge at room temperature for 10 min, and take the supernatant for testing. For detection of pyruvate content and lactate content, the content was detected using a Pyruvate Content Assay Kit or a Lactic Acid Content Assay Kit (Solarbio Science and Technology, Beijing, China), following the manufacturer’s instruction.

### Statistics

Statistical analyses were performed using GraphPad Prism 8.0 software (Chicago, IL, USA). Data are expressed as mean ± SD. Except where indicated, statistical significance was analyzed using two-tailed Student’s t-test and differences were considered statistically significant at *P* < 0.05.

## Results

### Sirt2 deficiency disrupts liver function and induces insulin resistance and reprogramming of glucose metabolism

The prevalence of obesity and T2DM has increased at an epidemic rate, a trend that continues unabated today [27]. Sirt2 inhibits T cell metabolism by targeting glycolysis, the tricarboxylic acid cycle, and fatty acid oxidation and other key enzymes to inhibit T cell metabolism, and T cells from Sirt2-deficient mice exhibit increased glycolysis and oxidative phosphorylation [28]. Therefore, we focused on Sirt2. We applied CRISPR-Cas9 technology to construct *Sirt2* knockout mice, and immunoblot analysis of mouse livers confirmed the absence of Sirt2 protein in *Sirt2*^*-/-*^ mice (Fig. S1A and 1B).

**Fig. 1:**
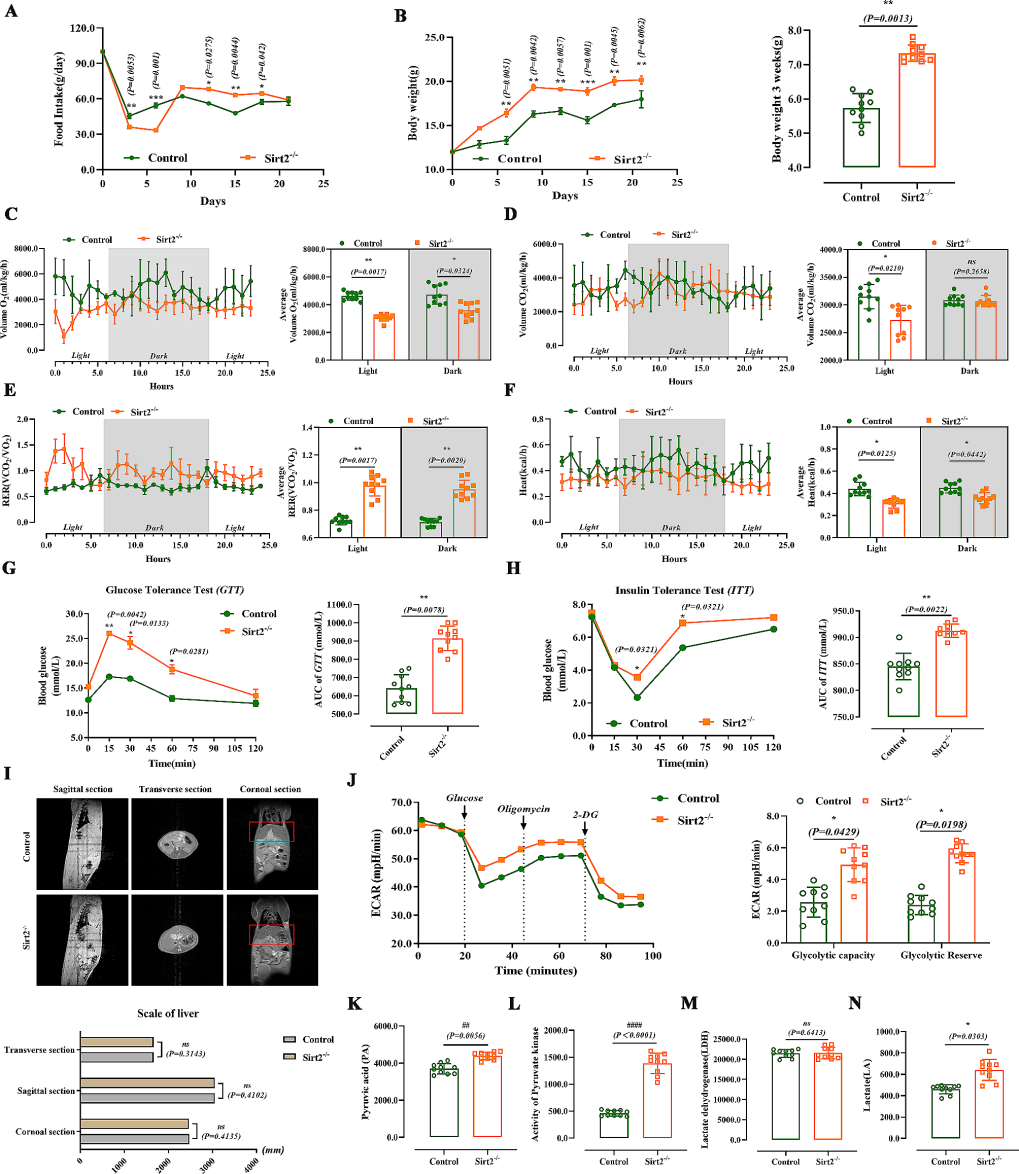
Sirt2 deficiency disrupts liver function and induces insulin resistance and reprogramming of glucose metabolism. **(A)** Food intake of mice in the control (*n* = 10/group) or *Sirt2*^*−/−*^ (*n* = 10/group) group at 8 weeks of normal feeding. **(B)** BW and weight change of mice in the two groups. (C-F) VO_2_**(C)**, VCO_2_**(D)**, respiration exchange ratio (RER) **(E)**, heat production **(F)** of control or *Sirt2*^*−/−*^ group (*n* = 6/group). **(G and H)** Blood glucose levels in control or *Sirt2*^*−/−*^ mice after 3 weeks of intensive feeding in GTT (G, left) and ITT (H, left) assays and the AUC of GTT (G, right) and ITT (H, right) assays were calculated (*n* = 6/group). **(I)** MRI of sagittal, transverse, and coronal sections of the liver (*n* = 6/group). **(J)** Extracellular acidification rate (ECAR) was determined by Seahorse XFe24 cell stress test (left). Glycolytic capacity and glycolytic reserve is shown on the right (*n* = 6/group). (K-N) Pyruvate content **(K)**, pyruvate activity **(L)**, lactate dehydrogenase activity **(M)**, and lactic acid content **(N)** were detected with corresponding kits (*n* = 6/group). Data are expressed as mean ± SD. Statistical significance was determined by the two-tailed Student’s *t*-test. ^*^P < 0.05; ^**^P < 0.01; ^***^P < 0.001; ns, not significant

To clarify the effect of *Sirt2* knockout on the energy metabolic processes in mice, *Sirt2*^*-/-*^ mice and controls were fed a normal diet from birth. We examined the basal indicators of food intake and body weight in both groups, and *Sirt2* knockout significantly increased the food intake (Fig. 1A) and body weight (Fig. 1B). This change in body weight may be achieved through increased energy intake, and we hypothesized that the effect of Sirt2 on body weight may also be due to reduced energy expenditure [29]. Therefore, we used metabolic cages and found that caloric consumption, VO_2_, VCO_2_, and heat were significantly lower in both groups of mice during the dark and light periods (Fig. 1C to F), and the RER was significantly higher, suggesting that knockout of *Sirt2* limited the energy consumption of the organism and that intake was greater than consumption, consistent with our hypothesis. This indicated that *Sirt2* knockout promoted primary obesity in mice. Therefore, we performed a glucose tolerance test (GTT) and insulin tolerance test (ITT) on *Sirt2*^*-/-*^ and control mice [30], and found that glucose tolerance and insulin resistance were more severe in *Sirt2*^*-/-*^ mice (Fig. 1G H); *Sirt2*^*-/-*^ mice also showed a slower decline and faster rebound of blood glucose, which implies possible insulin resistance. The report also indicated that liver-specific Sirt2 deficiency promoted insulin resistance, hepatic steatosis, and inflammation, while liver-specific Sirt2 overexpression reversed metabolic dysfunction [31]. Insulin resistance is closely associated with hepatic steatosis, nonalcoholic steatohepatitis [14]. In the present experiment, serum ALT and AST were increased in *Sirt2*^*-/-*^ mice, ALB levels were significantly lower, and there was a trend but no difference in TC elevation (Fig. S1D). ALT and AST were the most commonly used indicators to diagnose abnormal liver function [32]. These results suggested that knockout of *Sirt2* affected liver function in mice. Magnetic resonance examination is better for the diagnosis of substantial abdominal organs, especially liver diseases [33]. Our MRI results showed no significant difference in liver size between control and *Sirt2*^*-/-*^ mice (Fig. 1I and Fig. S1C), suggesting *Sirt2* knockout may not temporarily cause liver carcinogenesis, but had an effect on the physiological function of the liver [34]. However, the possible involvement of Sirt2 in the development of hepatocellular carcinoma was determined. Pathological examination revealed that the liver structure of *Sirt2*^*-/-*^ mice was loose. Analysis of the liver lobules by transmission electron microscopy (TEM) showed that the mitochondria in the livers of normal mice were regular in shape, mostly round or oval, and arranged neatly while in the *Sirt2* knockout mice, the mitochondrial structure was destroyed: mitochondria were deformed, cristae were broken, and the number was somewhat reduced (Fig. S1H). We found that *Sirt2* knockout aggravated the accumulation of reactive oxygen species (ROS) by dihydroethidium (DHE) staining, which showed increased red fluorescence, suggesting oxidative damage had occurred (Sup. Figure 1I). Increased apoptosis was observed by TUNEL staining (Fig. S1J). Overall, *Sirt2* knockout disrupted normal function.

To investigate why *Sirt2*^*-/-*^ mice were prone to metabolic problems, we next examined the extracellular acidification rate (ECAR) by Seahorse, a real-time assay of cellular bioenergetic metabolism [35], and found that *Sirt2* knockout increased glycolytic capacity (Fig. 1I). Recent studies have shown that SIRT2 in human stem cells regulated metabolic reprogramming during induction of pluripotency by targeting glycolytic enzymes [36, 37]. Knockout of *Sirt2* resulted in increased glycolysis. MicroRNA (miR)-200c-5p specifically targets SIRT2, downregulating its expression and enhancing acetylation of glycolytic enzymes and glycolysis, thereby promoting cell reprogramming [38]. By assaying pyruvate kinase enzyme activity, pyruvate content, lactate dehydrogenase activity, and lactate content in the glycolytic pathway, we found that *Sirt2* knockout increased pyruvate kinase activity, increased pyruvate content, and increased lactate content, with no significant change in lactate dehydrogenase (LDH) activity (Fig. 1K to N). Together, these results suggested that Sirt2 deletion affected liver function and that mice developed insulin resistance and induced reprogramming of glucose metabolism.

### Sirt2 induces deacetylation of K135 of PKM2 in a H187 site-dependent manner

**Fig. 2:**
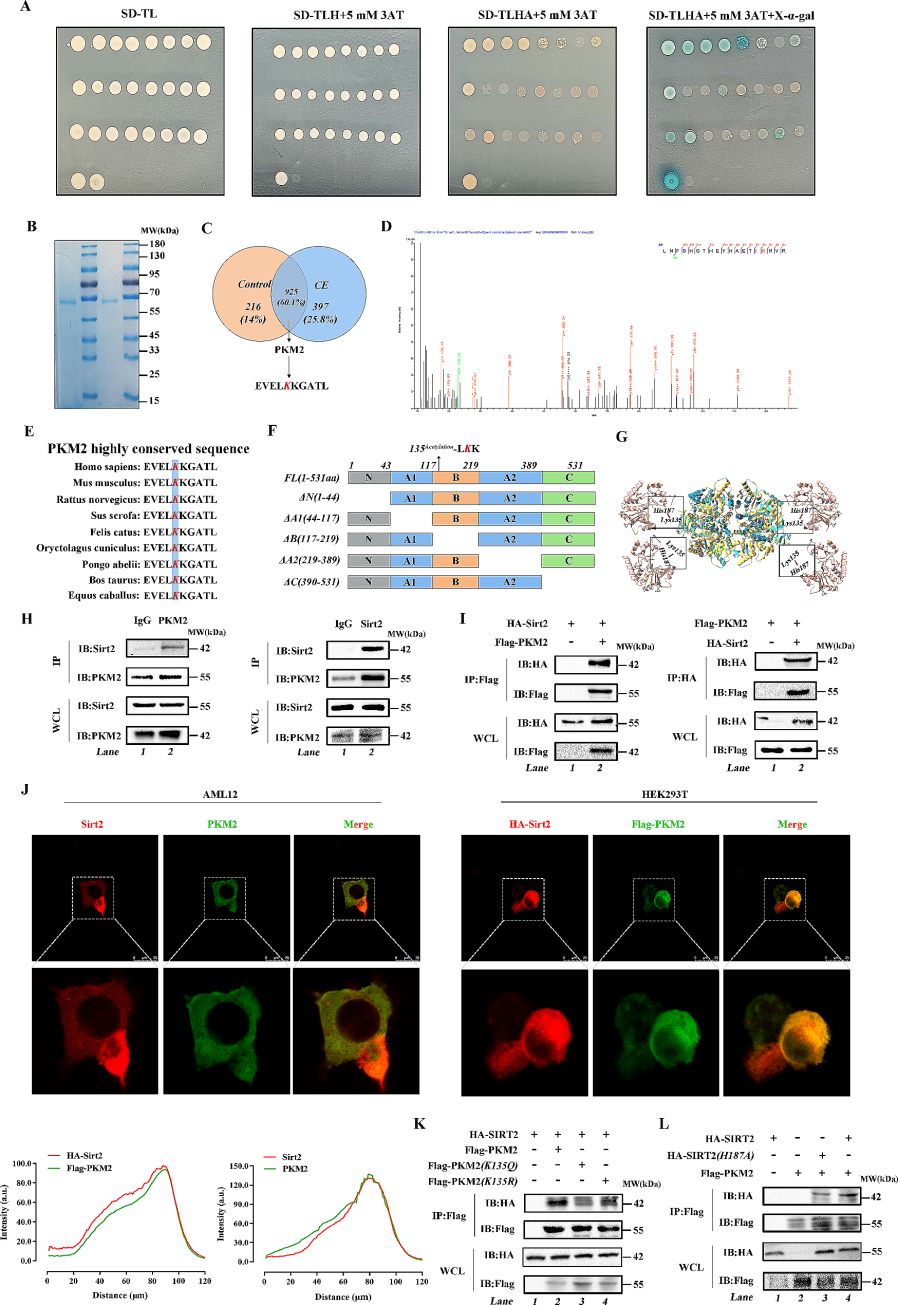
Sirt2 induces deacetylation of K135 of PKM2 in a H187 site-dependent manner. **(A)** Using the *Sirt2* gene constructed into the pGBKT7 vector as bait, a yeast two-hybrid mouse library was screened. After multiple reporter detection of positive clones, DNA sequencing and BLAST analysis, proteins interacting with PGBKT7-Sirt2 were identified. **(B)** Liver tissues of wild-type mice were analyzed by IP-MS. **(C and D)** Acetylation sites were analyzed by mass spectrometry. Venn diagram shows that PKM2 is conjectured as a common factor by acetylation modification omics **(C)**. PKM2 acetylation sites by mass spectrometry analysis **(D)**. **(E)** Sequences around K135 in PKM2 homologs of various species were compared. Acetylated lysine residues at PKM2-K135 are shown in bold red text. **(F)** Schematic diagram of various domain deletions of the PKM2 protein. **(G)** Molecular docking shows the binding domain of human PKM2 and Sirt2. **(H)** Endogenous PKM2 and Sirt2 co-immunoprecipitation using an anti-PKM2 antibody and detected with an anti-Sirt2 antibody (H, left). Endogenous Sirt2 and PKM2 co-immunoprecipitation using an anti-Sirt2 antibody and detected with an anti-PKM2 antibody (H, right). **(I)** Representative immunofluorescence staining of AML12 cells stained with Sirt2 antibody (red) and PKM2 antibody (green) (I, left) and fluorescence binding strength (I, right). Yellow indicates Sirt2 co-localization with PKM2 in AML12 cells (scale bar, 25 μm). **(J)** Co-immunoprecipitation with anti-Flag and anti-HA beads in HEK293T cells expressing HA-Sirt2 and Flag-PKM2 and detected with anti-Flag and anti-HA antibody. **(K)** Representative immunofluorescence staining of HEK293T cells stained with anti-Flag (green) and anti-HA (red) beads (K, left) and fluorescence binding strength (K, right). Yellow indicates HA co-localization with Flag in HEK293T cells (scale bar, 25 μm). **(L and M)** HEK293T cells were co-transfected with Flag-PKM2 (wild type) or Flag-PKM2 (K135Q/R) (mutant) plasmids and HA-Sirt2 was immunoprecipitated using an anti-Flag antibody and detected with an anti-HA antibody **(L)**. HEK293T cells were co-transfected with Flag-PKM2, HA-Sirt2, and HA-Sirt2 (H187Y), immunoprecipitated with an anti-Flag antibody, and detected with an anti-HA antibody **(M)**

To further investigated how Sirt2 regulates glucose metabolism disorder effects, we screened the proteins that interact with Sirt2 by yeast two-hybrid assay (Fig. 2A), and combined with the results of immunoprecipitation tandem mass spectrometry analysis, we found that PKM2, a key rate-limiting enzyme of the glycolytic pathway, had a reciprocal relationship with Sirt2 (Fig. 2B). Relevant studies on the association between Sirt2 and PKM2 are limited. Tandem mass spectrometry LC-MS/MS further confirmed these results and revealed that Lys135 (K135) was the major acetylation site of PKM2 (Fig. 2C and D); K135 was also an unreported acetylation site of PKM2. Previous studies have focused on deacetylation of SIRT2 at Lys 305 (K305) [39]. Notably, we previously identified Lys135 (K305) of PKM2 as a ubiquitination modification site. Through preliminary bioinformatics analysis, we found that K305 of PKM2 was highly conserved [40] and homologous in many species (Fig. 2E). To identify the protein structural domains of Sirt2 involved in PKM2 interactions, we performed molecular docking analysis (Fig. 2F and G). To investigate whether PKM2 interacted with Sirt2, we confirmed the in *vivo* interaction between Sirt2 and PKM2 in AML12 cells by endogenous co-immunoprecipitation (Co-IP) assay (Fig. 2H). In addition, we expressed Flag-PKM2 and HA-Sirt2 plasmids in HEK293T cells and performed pull-down assays, and observed PKM2-Sirt2 interaction (Fig. 2I). Immunofluorescence staining showed that Sirt2 and PKM2 co-localized in the cytoplasm, and similar results were obtained after co-transfection of HA-Sirt2 and Flag-PKM2 into HEK293T cells (Fig. 2J). To further determine the binding of Sirt2 to PKM2, we mutated K135 to glutamine (Q) and arginine (R), where glutamine mimics acetylated lysine and arginine mimics deacetylated lysine [41]. The Flag-tagged PKM2 wild-type and mutant plasmids were co-transfected with the HA-tagged Sirt2 plasmid into HEK293T cells, and the pull-down experiments showed that the binding of PKM2 to Sirt2 increased with the K135Q mutation, while the binding of HA to Flag decreased with the K135R mutation (Fig. 2K). Next, we mutated histidine 187 (H187) at the Sirt2 functional site to alanine (A) to make Sirt2 deficient in deacetylation [42], and found that the binding of dysfunctional Sirt2 increased with wild-type PKM2 (Fig. 2L), suggesting that K135 of PKM2 was modified by acetylation and Sirt2 was a deacetylase of PKM2. Sirt2 underwent deacetylation of K135 of PKM2 through a H187-enzyme active site-dependent manner.

### Inhibition of Sirt2 promotes expression of PKM2 and increases glycolytic capacity

**Fig. 3:**
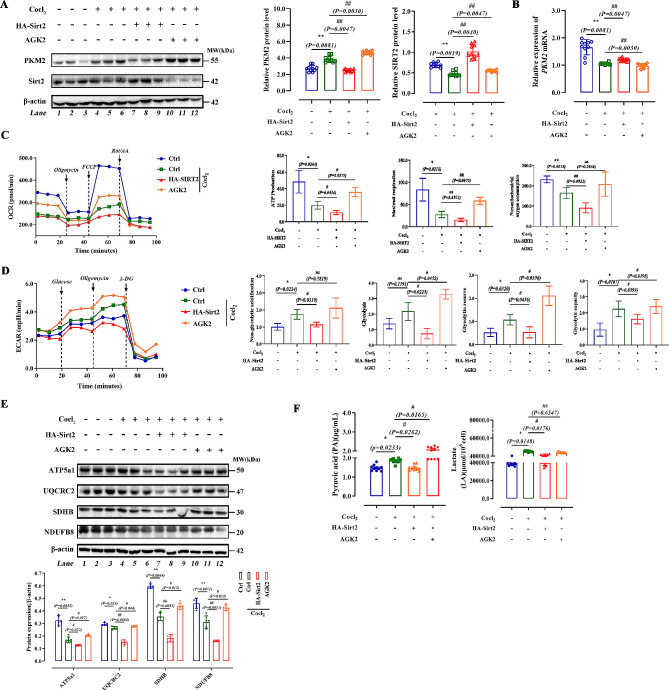
Inhibition of Sirt2 promotes expression of PKM2 and increases glycolytic capacity. **(A and B)** AGK2 and HA-Sirt2 were added to AML12 cells treated with CoCl_2_ (30 µmol) for 10 h. Western blotting of PKM2 and Sirt2 **(A).** Quantitative PCR analysis of PKM2. **(C and D)** Effects of CoCl_2_ on levels of oxygen consumption (OCR) and the extracellular acidification rate (ECAR) of different groups of primary hepatocytes. Levels of OCR **(C)** and ECAR were measured using the Seahorse XFe24 cell stress test **(D)** (*n* = 5/group). **(E)** Expression of ATP5a1, UQCRC2, SDHB, NDUFB8, and β-actin in four AML12 cell groups determined by western blotting. **(F)** Pyruvate acid (PA) content and lactate (LA) content in four AML12 cell groups were detected with the corresponding kit (*n* = 5/group). Data are expressed as mean ± SD. Statistical significance was determined by the two-tailed Student’s *t*-test. ^*^P < 0.05; ^**^P < 0.01; ^***^P < 0.001; ns, not significant

We further explored the role of Sirt2 in the deacetylation function of PKM2. Hypoxia has been reported to induce metabolic reprogramming of cells [43]. Therefore, we treated AML12 cells using the hypoxia mimetic cobalt chloride (CoCl2) (100 µmol) to generate a glucose metabolic reprogramming model. Then, a Sirt2 overexpression plasmid was added exogenously with the Sirt2 inhibitor AGK2 (10 µmol). We found that protein expression of PKM2 was reduced more significantly following overexpression of Sirt2 while levels of PKM2 were increased after the addition of AGK2 (Fig. 3A). The qPCR results indicated that overexpression of Sirt2 increased CoCl2-induced PKM2 gene expression in AML12 cells (Fig. 3B). We determined the effect of Sirt2 on mitochondrial oxygen consumption of cells under hypoxia by Seahorse assay and found that the level of oxidative phosphorylation decreased after CoCl2 treatment, but oxidative phosphorylation levels did not change significantly regardless of Sirt2 overexpression or inhibition (Fig. 3C). To clarify this phenomenon, we examined the expression of the components of mitochondrial respiratory complexes, and found that overexpression of Sirt2 was able to significantly inhibit the levels of mitochondrial respiratory chain complexes while inhibition of Sirt2 resulted in a significant increase in the mitochondrial respiratory chain complexes (Fig. 3D). Combined with the OCR results, we speculated that this elevated protein level was due to a compensatory response of the cells in terms of metabolism and function, allowing a new homeostasis to be established in vivo. To demonstrate whether Sirt2 affected lactate production, we also examined the extracellular acid production capacity and pyruvate content versus lactate content in each group of cells and found that inhibition of Sirt2 restored the extracellular acidification capacity (Fig. 3E and F). The above data indicated that inhibition of Sirt2 promoted the expression of PKM2, making its tetrameric form predominant and increasing the anaerobic oxidative capacity of cells.

### Deacetylation of K135 of Sirt2 promotes K48-linked ubiquitination-mediated degradation of PKM2

**Fig. 4:**
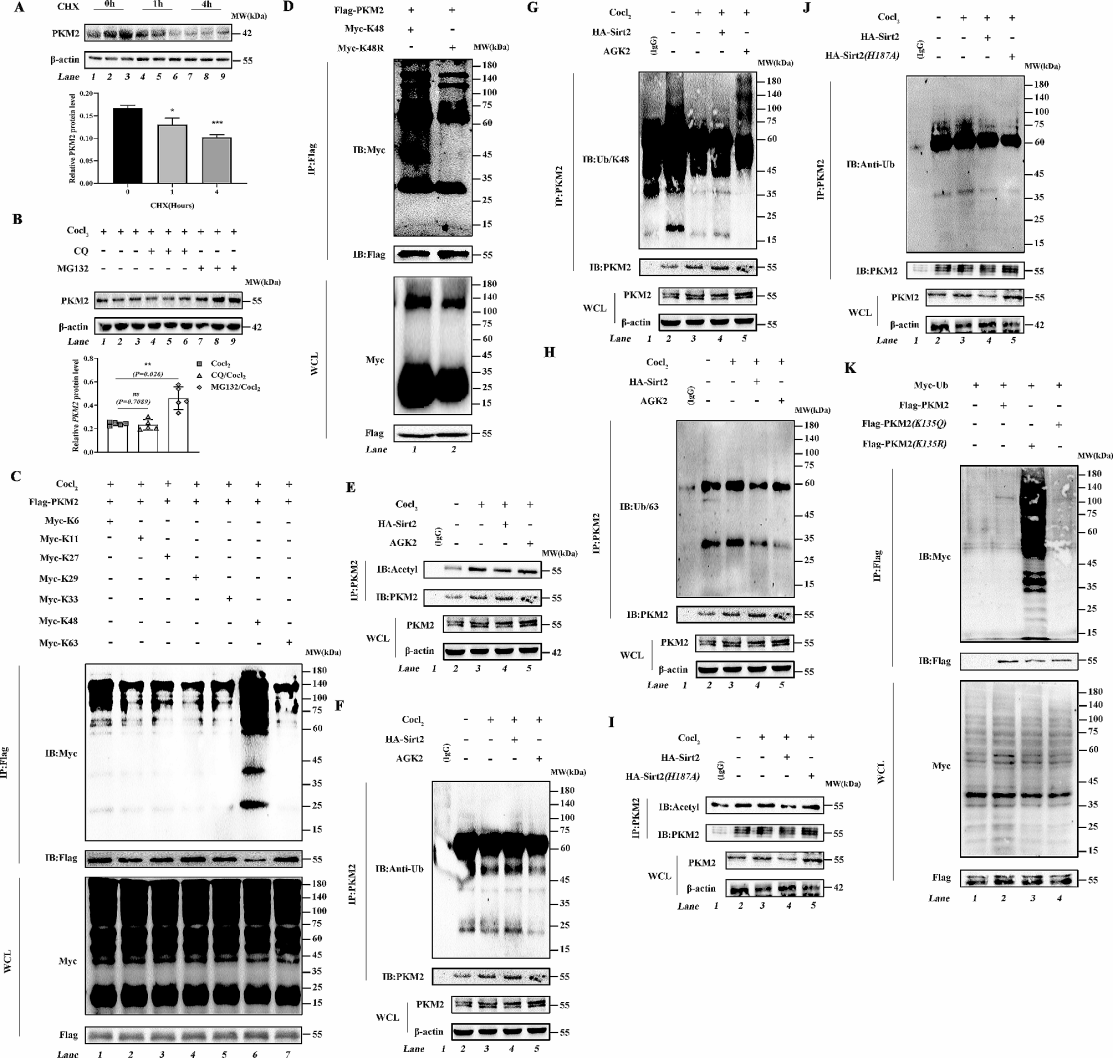
Deacetylation of K135 of Sirt2 promotes K48-linked ubiquitination-mediated degradation of PKM2. **(A)** PKM2 protein stability time course in AML12 cells following treatment with 100 µg/mL CHX for the indicated times. **(B)** AML12 cells after 10 h pretreatment with CoCl_2_ (30 µmol), and treatment with MG132 (10 µmol) for 2 h and CQ (25 µmol) for 2 h. Western blotting was used to assess protein levels of PKM2. **(C)** Flag-WT-PKM2 plasmid was transfected into HEK293T cells with different plasmids including K6, K11, K27, K29, K33, K48, and K63. Lysates were immunoprecipitated with anti-Flag antibody and blotted with anti-Myc antibody. **(D)** Flag-WT-PKM2 plasmid was transfected into HEK293T cells with different plasmids including K48 and K48R. Lysates were immunoprecipitated with anti-Flag antibody and blotted with anti-Myc antibody. **(E–H)** Co-immunoprecipitation experiments of control, CoCl_2_, CoCl_2_/AGK2, and CoCl_2_/HA-Sirt2 cells using an anti-PKM2 antibody for IP and anti-acetyl-lysine **(E**), anti-ubiquitin **(F)**, anti-K48-linked ubiquitin chain **(G)**, and K63-linked ubiquitin chain **(H)** antibodies for immunoblotting. **(I and J)** After 10 h pretreatment with CoCl_2_, Co-IP experiments of AML12 cells expressing HA-Sirt2 or mutant plasmid (HA-Sirt2/H187Y) were performed using an anti-PKM2 antibody for IP and anti-acetyl-lysine **(I)** and anti-ubiquitin (J) antibodies for immunoblotting. **(K)** Myc-ubiquitin plasmid was transfected into HEK293T cells expressing Flag-PKM2, Flag-PKM2(K135Q), and Flag-PKM2(K135R) plasmids. Lysates were immunoprecipitated with anti-Flag antibody and blotted with anti-Myc antibody. Data are expressed as mean ± SD. Statistical significance was determined by the two-tailed Student’s *t*-test. ^*^P < 0.05; ^**^P < 0.01; ^***^P < 0.001; ns, not significant

To clarify the mode of regulation of PKM2 by Sirt2, we treated AML12 cells with 10 µM cycloheximide (CHX) for 0, 1, and 4 h. AML12 cells showed significantly reduced PKM2 levels after 4 h of CHX treatment; furthermore, the effect of Sirt2 on PKM2 was proteasome-mediated because 20 µM MG132 completely reversed degradation of PKM2 (Fig. 4A). Significant PKM2 protein degradation was still observed in cells treated with the lysosomal inhibitor chloroquine (CQ) (Fig. 4B). These results suggested that Sirt2 may inhibit degradation of PKM2 by the ubiquitin-proteasome pathway. Because different forms of ubiquitin chains play different roles in regulating protein function [44, 45], we transfected Flag-PKM2 expression plasmids into HEK293T cells together with a ubiquitin plasmid containing only a single lysine residue. We found that PKM2 had the highest level of ubiquitination at K48 in the CoCl2 construct model (Fig. 4C). We thus constructed a plasmid with mutation of the K48 site (K48R) to reduce the degree of ubiquitination (Fig. 4D). Mutation of K48 largely reduced ubiquitination of PKM2. We next examined acetylation and ubiquitination levels of PKM2, and we found that, by immunoprecipitation, acetylation of PKM2 could inhibit ubiquitination (Fig. 4E-H). We then mutated H187 of Sirt2 to tyrosine (Y), which resulted in impaired deacetylation and an increase in acetylation and a decrease in ubiquitination (Fig. 4I and J). Cotransfection of the total ubiquitin plasmid with wild-type and mutant PKM2 plasmids into HEK293T showed that mock deacetylation of K135 of PKM2 increased ubiquitination of PKM2 (Fig. 4K). These data suggested that deacetylation of K135 of PKM2 by Sirt2 promoted K48 ubiquitin chain-mediated degradation and thus inhibited protein stability.

### Knockdown of Sirt2 disrupts liver function in mice under metabolic stress

**Fig. 5:**
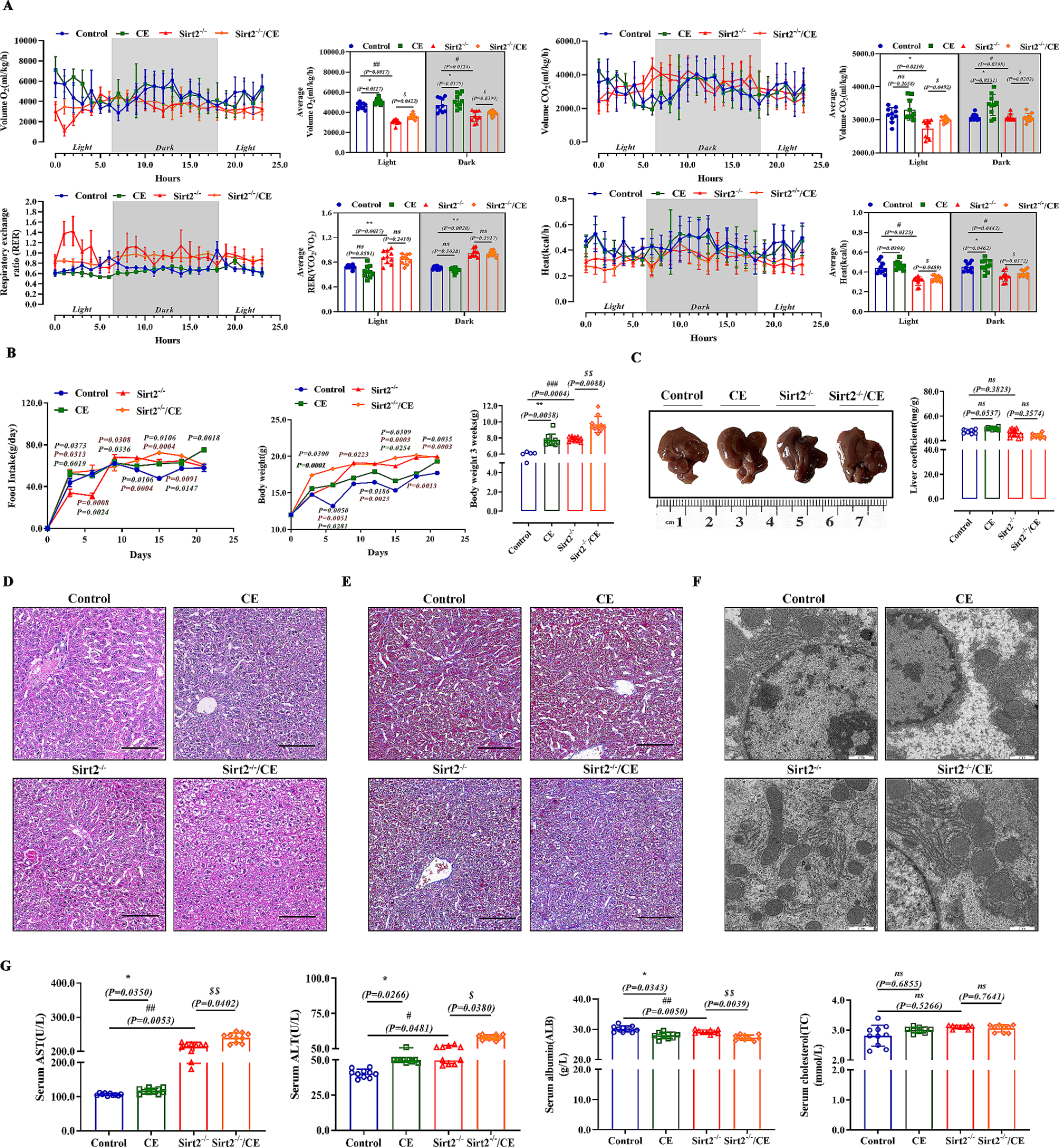
Knockdown of Sirt2 disrupts liver function in mice under metabolic stress. *Sirt2*^*-/-*^ and WT mice aged 5 weeks were divided into four groups (Control, CE, *Sirt2*^*-/-*^, and *Sirt2*^*-/-*^/CE) and fed diets for 3 weeks. **(A)** VO_2_, VCO_2_, respiration exchange ratio (RER), and heat production of each group (*n* = 6/group). **(B)** Food intake of mice and body weight (BW) and weight change of mice in each group (*n* = 10/group) after 8 weeks of normal feeding. **(C)** Representative images and liver-to-body weight ratios. **(D-F)** Representative H&E **(D)** and Masson **(E)** staining, and TEM **(F)** images of liver sections. **(G)** Serum AST, ALT, ALB, and TC levels (*n* = 10/group). Data are expressed as mean ± SD. Statistical significance was determined by the two-tailed Student’s *t*-test. ^*^P < 0.05; ^**^P < 0.01; ^***^P < 0.001; ns, not significant

Using in vitro experiments, we initially clarified the role of Sirt2 in glycolysis. To further investigate the effect of Sirt2 on liver glycolysis in mice, we constructed *Sirt2* knockout mice. Chronic cold exposure (CE) was applied to wild-type and *Sirt2*^*-/-*^ mice to construct a metabolic stress model and mobilize energy in vivo. By metabolic cage assay we found that oxygen consumption (VO_2_) and carbon dioxide production (VCO_2_) were significantly higher in the CE group during both daytime and dark periods, with no change in respiratory function exchange ratio (RER) (Fig. 5A), consistent with previous results [46]. The same was true for *Sirt2*^*-/-*^/CE group mice. In addition, CE significantly promoted caloric consumption in both control and *Sirt2*^*-/-*^ group mice. Treatment significantly increased the food intake and body weight of mice in the control and *Sirt2*^*-/-*^groups (Fig. 5B), suggesting that external stress may have contributed to weight gain by reducing energy metabolism in mice, which was exacerbated by *Sirt2* knockout. We performed anatomical morphological assessment of the mouse liver; no significant change in liver size and liver to body weight ratio was observed (Fig. 5C). Histological analysis (hematoxylin and eosin and Masson staining) showed that the liver structure of WT mice was loosened by cold stimulation, and knockdown of Sirt2 led to more severe structural destruction of the liver, as shown by the unclear structure of liver lobules and occasional cellular infiltration, suggesting abnormal liver function (Fig. 5D and E). TEM showed that cold aggravated mitochondrial structure after Sirt2 knockdown; mitochondrial cristae were broken and reduced in number (Fig. 5F). Cold significantly increased serum AST and ALT levels and significantly decreased serum ALB levels in control group mice; and these indicators were also significant in *Sirt2*^*-/-*^ mice while cholesterol levels did not show significant changes in the four groups (Fig. 5G). Taken together, the results suggested that Sirt2 severely affected external cold stimulation on mouse liver function, and we speculated that this also exacerbated the metabolic burden on glucose metabolism in the liver in a cold environment.

### Metabolic stress exacerbates the effects of Sirt2 deletion on metabolic reprogramming

**Fig. 6:**
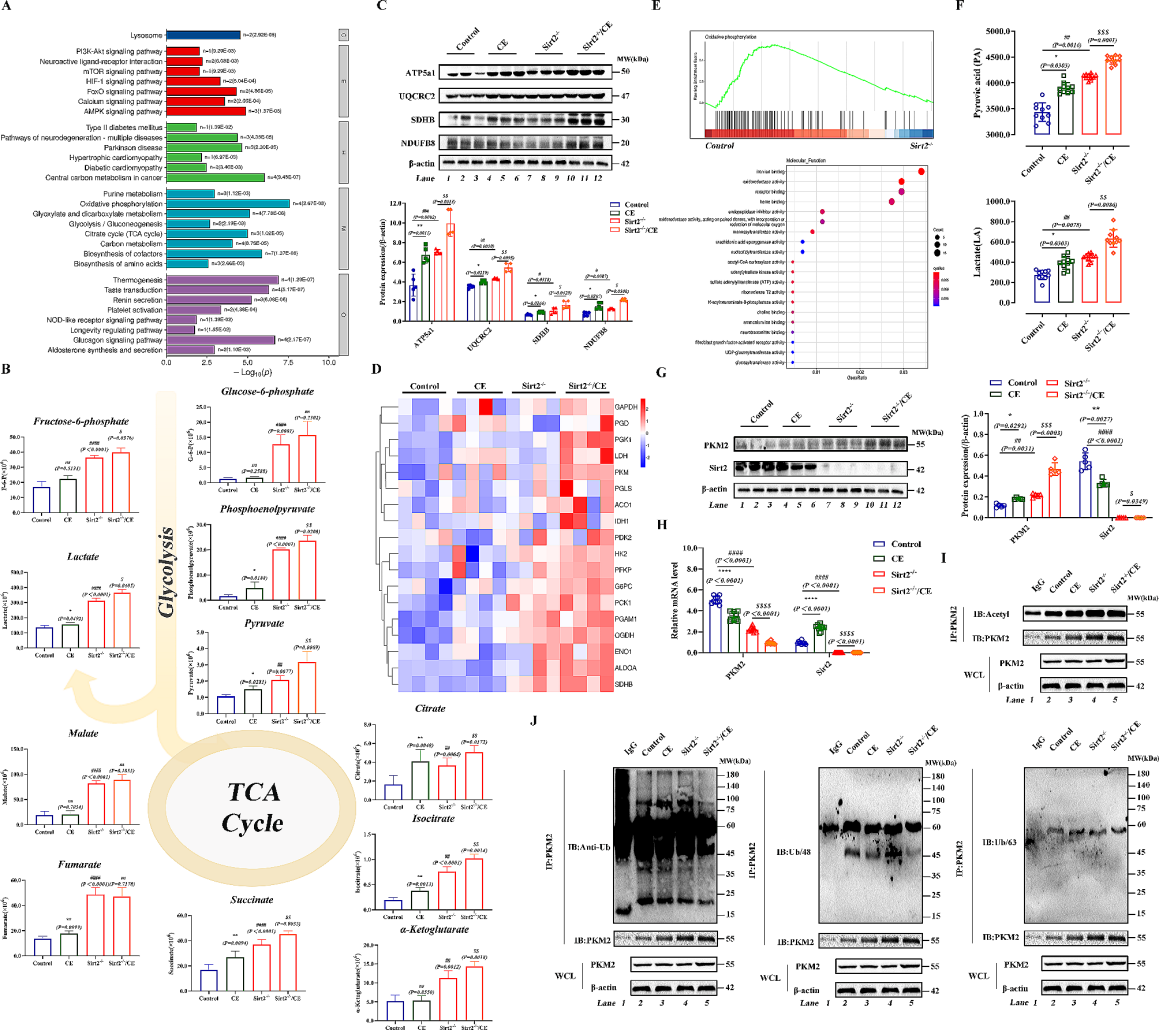
Metabolic stress exacerbates the effects of Sirt2 deletion on metabolic reprogramming. **(A and B)** Relative quantitative analysis of energy metabolites by targeted metabolomics. KEGG analysis was performed for energy metabolites **(A)** and expression levels of the metabolites were detected **(B)**. **(C)** Expression of ATP5a1, UQCRC2, SDHB, NDUFB8, and β-actin in four different groups determined by western blot analysis. **(D)** Heat map of differentially expressed genes of WT and Sirt2 mice upon cold exposure by RNA-sequencing. **(E)** Gene set enrichment analysis (GSEA) and Gene Ontology (GO) functional annotation by RNA-sequencing. **(F)** PA and LA contents were detected with the corresponding kit (*n* = 10/group). **(G)** Western blot analysis of PKM2 and Sirt2. **(H)** Quantitative PCR analysis of PKM2 and Sirt2. **(I and J)** Using an anti-PKM2 antibody for IP and anti-acetyl-lysine **(I)**, anti-ubiquitin (J, left), anti-K48-linked ubiquitin chain (J, middle), and K63-linked ubiquitin chain (J, right) antibodies for immunoblotting. Data are expressed as mean ± SD. Statistical significance was determined by the two-tailed Student’s *t*-test. ^*^P < 0.05; ^**^P < 0.01; ^***^P < 0.001; ns, not significant

Kyoto Encyclopedia of Genes and Genomes (KEGG) pathway enrichment analysis showed differences in multiple pathways including glycolysis, tricarboxylic acid (TCA) cycle, and oxidative phosphorylation (Fig. 6A). During glycolysis, we found elevated levels of some major metabolites in the CE and *Sirt2*^*-/-*^ groups, such as glucose 6-phosphate (glucose-6p), fructose 6-phosphate (frucotose-6p), PEP, and pyruvate. In addition, the *Sirt2*^*-/-*^/CE group exhibited higher levels of metabolites. Lactate levels showed the same trend as other metabolites. Elevated levels of glucose, pyruvate, and lactate not only maintained the energy required by the body, but also produced large amounts of the metabolite pyruvate, indicating an enhanced anaerobic glycolytic process and metabolic reprogramming. The primary metabolites of the TCA cycle showed a consistent trend with glycolytic products (Fig. 6B). The levels of mitochondrial respiratory complexes were significantly increased in both CE and *Sirt2*^*-/-*^ groups (Fig. 6C). Our RNA sequencing data has been uploaded to the NCBI database with GEO data number GSE229161, and the results indicated that the expression levels of related genes in metabolic pathways showed the same trend as metabolomics (Fig. 6D). GSEA analysis revealed that differential genes were correlated with oxidative phosphorylation process, KEGG pathways were enriched in metabolism-related pathways (Fig. 6E), and pyruvate and lactate contents were consistent with metabolomics results (Fig. 6F). Taken together, our results suggested that low temperature accelerated the glycolytic process following knockdown of Sirt2. In addition, the CE group showed a high level of PKM2 and *Sirt2* knockout promoted protein levels of PKM2 (Fig. 6G). mRNA levels showed opposite trends as the protein levels (Fig. 6H). Thus, Sirt2 affects protein degradation of PKM2 but has no effect on protein biosynthesis. Finally, we explored the function of Sirt2 in PKM2 deacetylation. By co-immunoprecipitation, we found that acetylation of PKM2 could promote its ubiquitination following CE, and knockdown of Sirt2 further promoted acetylation of PKM2, accelerated its ubiquitination (Fig. 6I), and affected modification through K48-linked ubiquitin chains (Fig. 6J). The above results suggested that metabolic stress exacerbated the effects of Sirt2 deletion on liver function and reprogramming of glucose metabolism.

### Sirt2 supplementation alleviates the disruption of liver function and metabolic disorders under metabolic stress

**Fig. 7:**
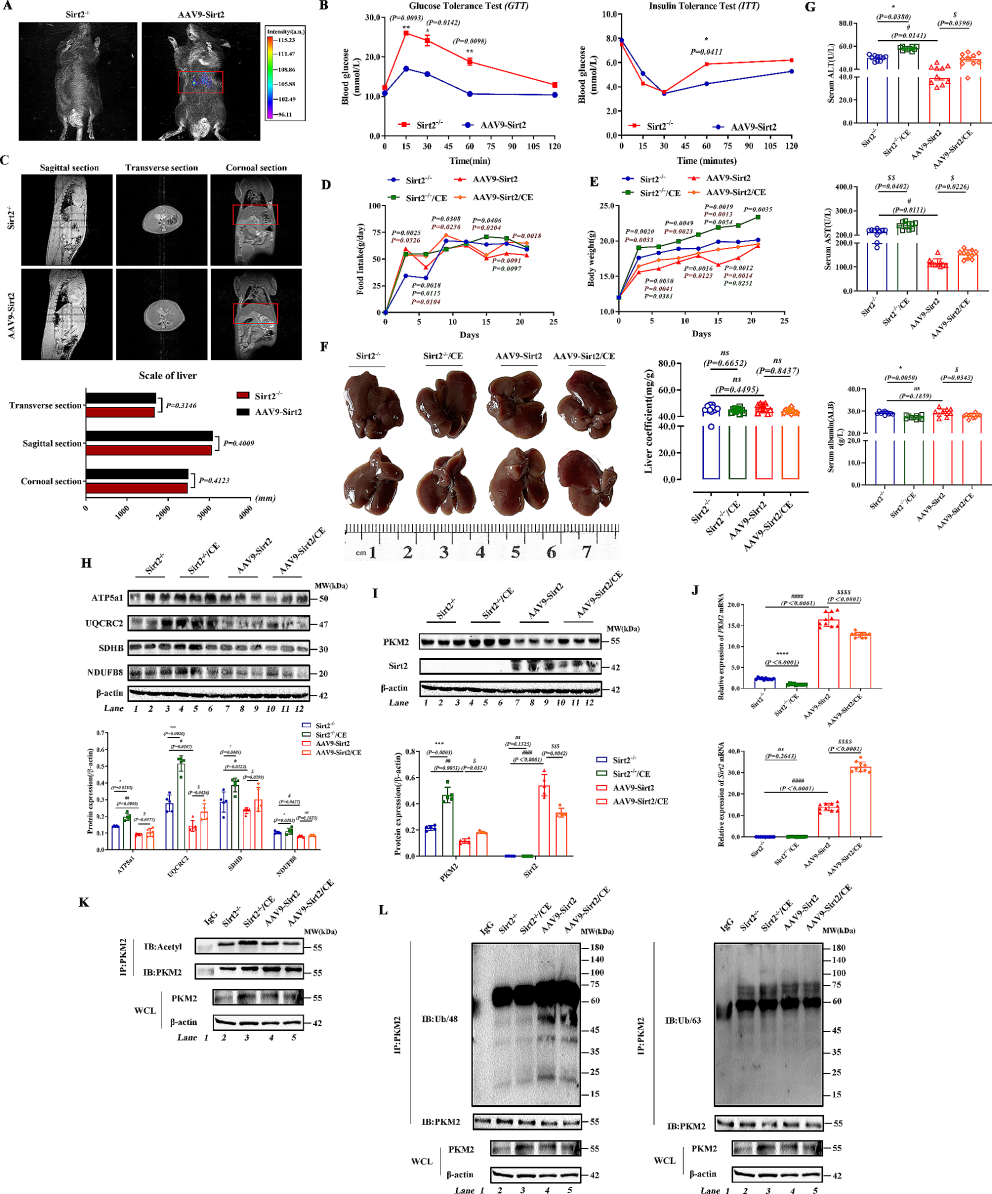
Sirt2 supplementation alleviates the disruption of liver function and metabolic disorders under metabolic stress. *Sirt2*^*-/-*^ mice were injected with the liver specific adeno-associated virus HBAAV9-TBG-m-Sirt2-Luc and cold exposure was administered to the *Sirt2*^*-/-*^ and AAV9-*Sirt2* groups. **(A)** The *Sirt2* gene in liver was observed by visible light imaging in vivo. **(B)** Blood glucose levels in control or *Sirt2*^*-/-*^ mice after 3 weeks of intensive feeding as determined by GTT and ITT (B, left) assays (*n* = 6/group). **(C)** MRI examination of sagittal, transverse, and coronal sections of the liver **(D)** Food intake of mice in each group after 8 weeks of normal feeding. **(E)** BW and weight change in the four groups (*n* = 10/group). **(F)** Representative images and liver-to-body weight ratios. **(G)** Serum AST, ALT, ALB, and TC levels (*n* = 10/group). **(H)** Expression of ATP5a1, UQCRC2, SDHB, NDUFB8, and β-actin in four different groups determined by western blotting. **(I)** Western blot analysis of PKM2 and Sirt2. **(J)** Quantitative PCR analysis of PKM2 and Sirt2. **(K–L)** Using an anti-PKM2 antibody for IP and anti-acetyl-lysine **(K)**, anti-K48-linked ubiquitin chain and K63-linked ubiquitin chain **(L)** antibodies for immunoblotting. Data are expressed as mean ± SD. Statistical significance was determined by the two-tailed Student’s *t*-test. ^*^P < 0.05; ^**^P < 0.01; ^*^**P < 0.001 ***P < 0.001; ns, not significant

Finally, we constructed the AAV9 viral system for validation, we measured the expression level of AAV9 virus in mice by in vivo imaging technology, and the results show that the virus is only specifically expressed in the liver (Fig. 7A). We found that glucose tolerance and insulin resistance were gradually restored following *Sirt2* injection in the knockout mice (Fig. 7B; Fig. S2A), and liver MRI results showed no abnormal liver morphology in the second group of mice (Fig. 7C). We then measured ECAR levels by Seahorse, which showed glycolytic capacity was reduced after Sirt2 supplementation (Fig. S2B), consistent with our previous experimental results. Similarly, we applied cold exposure to mice, and found that supplementation of Sirt2 significantly reduced the feeding and body weight of mice (Fig. 7D and E; Fig. S2C). There was no change in liver size or liver weight ratio in the four groups (Fig. 7F) while the levels of ALT, AST, and ALB were alleviated after supplementation of Sirt2 (Fig. 7G). Histological analysis verified the above results (Fig. S2D). Pyruvate and lactate contents in the AAV-Sirt2 group showed a significant decrease, but the levels increased after cold exposure (Fig. S2E). Protein expression levels of the mitochondrial respiratory chain complex showed a similar trend (Fig. 7H). We examined protein levels of PKM2, and found that the levels of PKM2 in the AAV-Sirt2 group were significantly reduced compared to the *Sirt2*^-/-^ group (Fig. 7I); the gene level showed an opposite trend (Fig. 7J). We verified that Sirt2 regulated the deacetylation function of PKM2, and found that knockdown of Sirt2 with CE promoted acetylation of PKM2 (Fig. 7K), accelerating its ubiquitination via specific K48-linked ubiquitin chains, while acetylation was decreased and ubiquitination was increased after AAV9-Sirt2 injection (Fig. 7L). This validated the previous findings.

## Discussion

Obesity increases the risk of systemic insulin resistance and T2DM, a global public health crisis that affects more than 500 million adults worldwide [47]. In this study, we determined the protective role of Sirt2 in liver injury, obesity-related insulin resistance, and glucose metabolism. First, expression of Sirt2 was significantly reduced in obese and diabetic patients. Second, Sirt2 deficiency resulted in significant primary obesity and insulin resistance accompanied by hepatic metabolic dysfunction. Previous studies specified that hepatic overexpression of Sirt2 improved insulin sensitivity, oxidative stress, and mitochondrial dysfunction in obese mice [30]. Therefore, targeting Sirt2 may be a potential therapeutic strategy for the treatment of related metabolic disorders.

In this study, we identified PKM2 as a substrate of Sirt2, a key metabolic enzyme that catalyzes the final step of the glycolytic reaction, through a yeast two-hybrid assay and immunoprecipitation tandem mass spectrometry analysis [26]. Protein acetylation has become a key post-translational modification in cellular metabolism, as almost every glycolytic and TCA cycle enzyme undergoes acetylation [48]. We also identified multiple acetylation sites of PKM2 by acetylation histology: K89, K135, and K305. It has been previously shown that PKM2 activity was impacted by altered lysine acetylation [49], with PKM2 acetylated by p300 at K433, which prevented activation of PKM2 by interfering with FBP binding and promoted nuclear accumulation of PKM2 and protein kinase activity [50, 51]. It is true that the relationship between Sirt2 and PKM2 has been reported, but in this experiment, we identified a novel PKM2 acetylation modification site, K135, which was undiscovered and unstudied, and furthermore, the ubiquitination modification histology in our lab in the pre-laboratory showed that the K135 site is also a ubiquitination modification site of PKM2, which enables us to explore whether there is a competitive/promotional relationship between the acetylation of PKM2 modification and whether there is a competitive/promotional relationship between ubiquitination modifications. Our study also showed that Sirt2 underwent deacetylation modification of K135 of PKM2 in a H187-dependent manner. In vitro experiments confirmed that in a CoCl_2_-induced reprogramming model of AML12 glucose metabolism, Sirt2 reduced the acetylation of PKM2-K135 and increased the ubiquitination of K48 of PKM2. K48-linked ubiquitin chains normally lead to ubiquitin proteasome pathway degradation of the modified substrate [51], our results also suggested that the proteasome pathway mediated the subsequent PKM2 degradation. It has been shown that the function of PKM2 was also dependent on post-translational modifications, directing activity through a mechanism that favored tetrameric structure [2]. Under normal conditions, the tetrameric form of PKM2 has high glycolytic activity and glucose is converted to pyruvate for energy production. Lactic acid increases the level of lactonization of PKM2, inhibits its tetramer to dimer transition, promotes its pyruvate kinase activity, and reduces nuclear distribution [52]. In the present study, Sirt2 was shown to inhibit expression of PKM2, promoting glucose metabolism and lactate production and increasing the glycolytic capacity of cells. This is thus a novel mechanism by which acetylation directs the function of PKM2.

To investigate the effect of Sirt2 on liver glycolysis in mice, we constructed *Sirt2* knockout mice. Sirt2 gene polymorphisms have been reported to be associated with the maintenance of glucose homeostasis. Watanabe reported that *Sirt2* downregulation resulted in a slight increase in blood glucose levels and significant hyperinsulinemia [53]. Lantier also showed that severe insulin resistance occurred in the liver of *Sirt2* knockout rats fed a high-fat diet [28]. Our results found that Sirt2 deficiency led to impaired glucose tolerance and insulin resistance and that KO mice had a higher diet and body weight but reduced caloric expenditure and developed significant primary obesity with concomitant hepatic metabolic dysfunction [54]. After we gave mice cold stimulation to construct a metabolic stress model to mobilize in vivo energy. Under normal conditions, an appropriate low temperature environment ameliorates metabolic impairment, for example, cold exposure significantly promotes glucose uptake from brown fat in hormonal mice and promotes metabolism [18]. Previous studies have shown that in order to coordinate beige adipocyte plasticity and enhance adaptation to low temperature, HIFα was activated by microenvironmental alterations and alleviated metabolic stress by regulating cellular pathways to alleviate metabolic stress [55]. We therefore focused our attention on how Sirt2 regulates body glucose metabolism in a state of metabolic stress, and when we gave wild-type mice and Sirt2 KO mice intermittent hypothermic stimulation for three weeks, mice fed to meet increased metabolic demands and thermoregulation increased and the mice showed weight gain accompanied by hepatic metabolic dysfunction, which was made more pronounced by the knockout of *Sirt2*. The hypothermic environment stimulates energy expenditure, increases energy intake in thermostatic animals, and stimulates glucose translocation to the cell and glucose metabolism [32]. Research showed that the level of glycolysis within the BAT is elevated under cold conditions, and we found by metabolomics that the levels of major metabolites during glycolysis under metabolic stress increased, and in the previous part of this experiment, we clarified that *Sirt2*^−/−^ induced reprogramming of glucose metabolism, and that Sirt2 downregulation led to hyperacetylation of glycolytic enzymes and enhanced glycolysis is also a phenomenon that has been identified by many scholars, and here, we are more certain that metabolic stress exacerbates the negative regulatory mechanism of Sirt2 on glycolysis. In vitro experiments initially determined that Sirt2 regulates the expression of PKM2, so does PKM2 play a functional role in the negative regulation mechanism of glycolysis by Sirt2 under metabolic stress? The results showed that knockdown of Sirt2 under low temperature environment further promoted acetylation of PKM2 and inhibited its ubiquitination, and affected the modification through specific K48 ubiquitin chain. Mechanistically, this suggests that the deletion of Sirt2 in a state of metabolic stress exacerbates impaired substance metabolism, which in turn severely affects the hepatic metabolic burden of mice, promotes PKM2 protein degradation via the ubiquitin-proteasome pathway, and prevents the conversion of cytoplasmic PKM2 to the nucleus, thereby affecting the PKM2-mediated glycolytic process. To prove this retrospectively, we constructed an AAV9 viral expression system with a hepatocyte-specific TBG promoter to verify this, and we found that Sirt2 back-complementation reversed the changes in the glycolytic process brought about by Sirt2 deletion, and the results here also indicate our conclusion.

## Conclusions

**Fig. 8:**
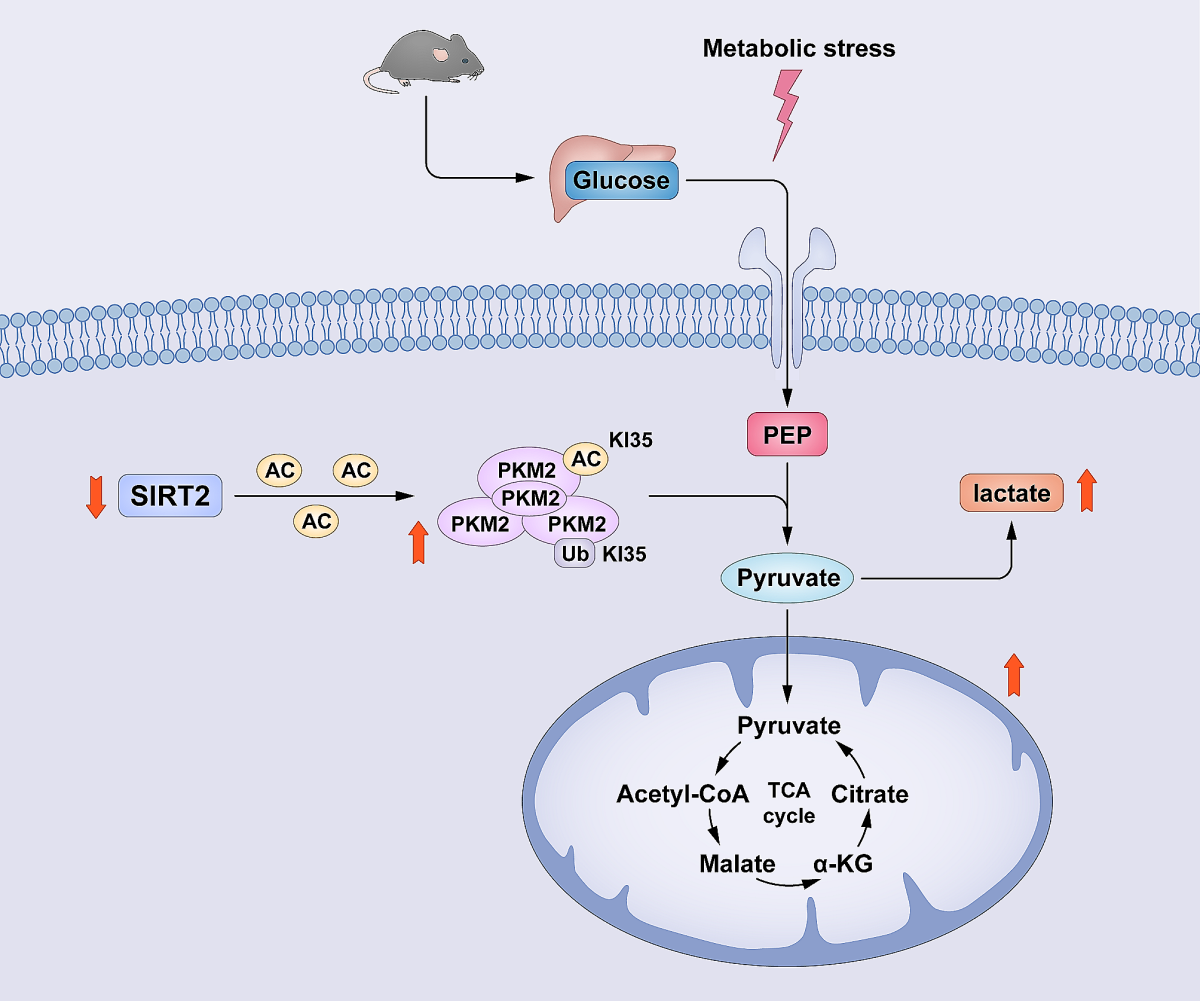
The role of NAD-dependent deacetylase sirtuin-2 in liver metabolic stress through regulating Pyruvate kinase M2 ubiquitination. Sirt2 disrupts liver function in mice under metabolic stress, exacerbating the metabolic burden on the liver and impacting glucose metabolism. Sirt2 has an effect on lysine 135 of PKM2 through a histidine 187 enzyme active site-dependent site acetylation modification occurs and reduces ubiquitination of the K48 site ubiquitin chain of PKM2 thereby altering the adaptation to the metabolic stress response and ultimately regulating the glycolytic process

In summary, here we found that Sirt2 deletion leads to impaired glucose tolerance and insulin resistance, induced primary obesity; Sirt2 severely disrupts liver function in mice under metabolic stress, exacerbating the metabolic burden on the liver and impacting glucose metabolism. Sirt2 has an effect on PKM2 K135 through H187 site acetylation modification occurs and reduces ubiquitination of the K48 site of PKM2 thereby altering the adaptation to the metabolic stress response and ultimately regulating the glycolytic process (shown in Model in Fig. 8). Therefore, Sirt2 may regulate hepatic glucose metabolism and provide a new target for future clinical treatment of metabolic diseases.

### Electronic supplementary material

Below is the link to the electronic supplementary material.

Supplementary Material 1

Supplementary Material 2

## Data Availability

Data generated in this study are publicly available in the GEO repository (GSE229161). We also utilized publicly accessible RNA-seq data from GEO repositories GSE474, GSE36297, GSE20966, and GSE1010.
