# Supplementary figures and images for: The role of NAD-dependent deacetylase sirtuin-2 in liver metabolic stress through regulating pyruvate kinase M2 ubiquitination
Source: J Transl Med. 2024 Jul 14;22:656. doi: 10.1186/s12967-024-05435-w (PMC11247741; doi:10.1186/s12967-024-05435-w)

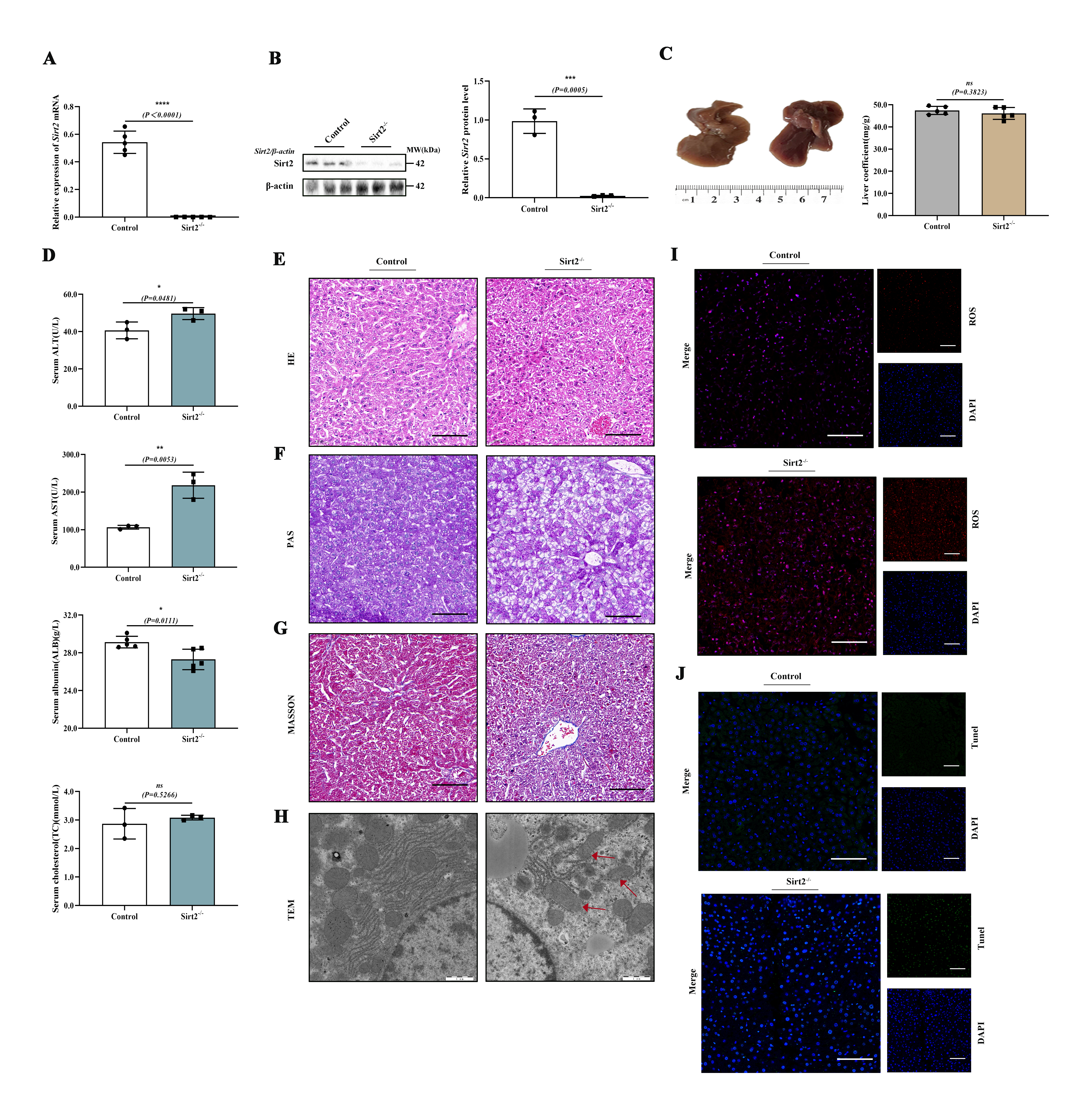

Supplement: Supplementary file 1 — Supplementary Material 1 [file 12967_2024_5435_MOESM1_ESM.tif]

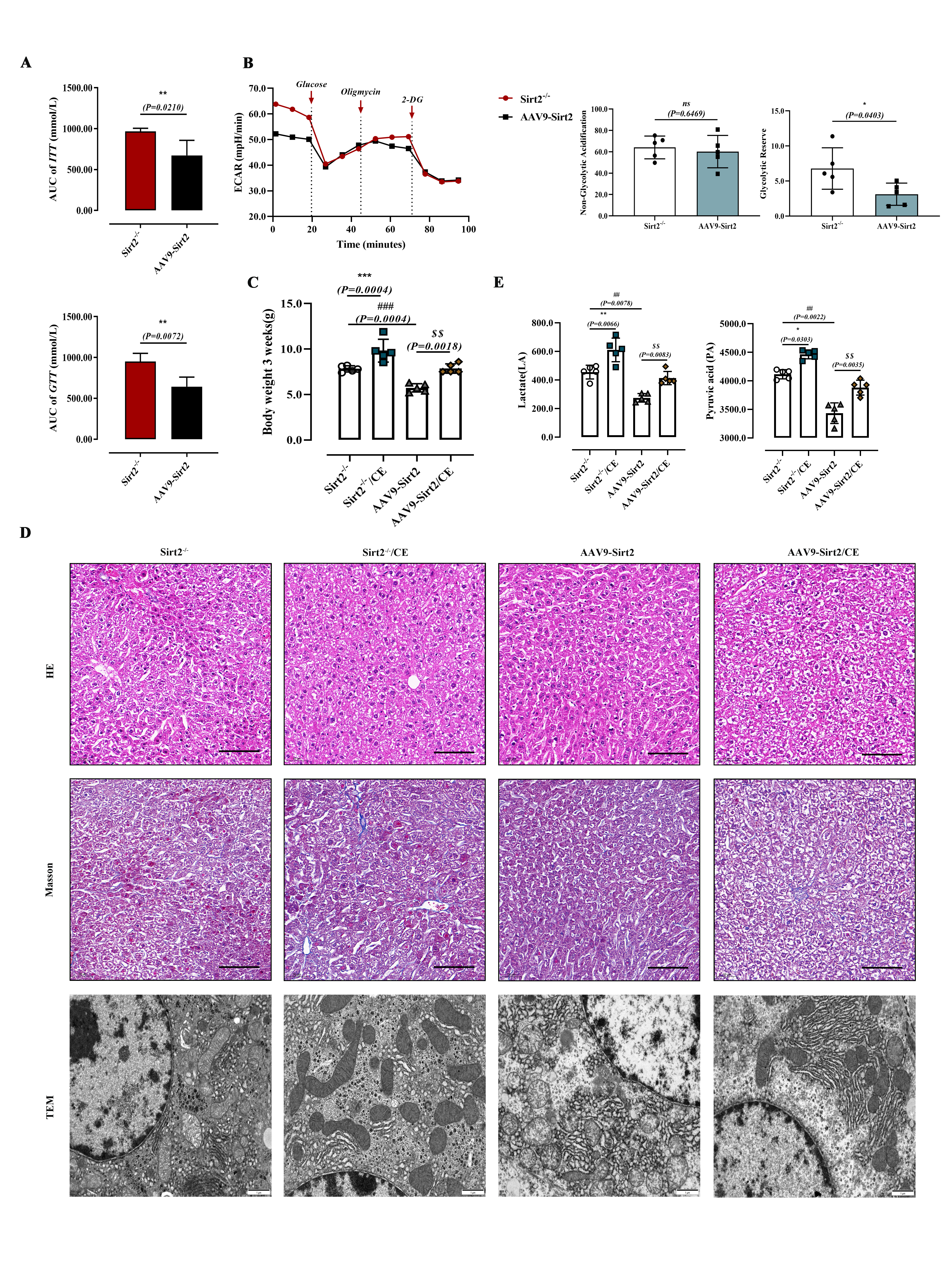

Supplement: Supplementary file 2 — Supplementary Material 2 [file 12967_2024_5435_MOESM2_ESM.tif]
